# High Prevalence Rate of Microbial Contamination in Patient-Ready Gastrointestinal Endoscopes in Tehran, Iran: an Alarming Sign for the Occurrence of Severe Outbreaks

**DOI:** 10.1128/spectrum.01897-22

**Published:** 2022-09-29

**Authors:** Hamidreza Houri, Hamid Asadzadeh Aghdaei, Sepideh Firuzabadi, Babak Khorsand, Fatemeh Soltanpoor, Maedeh Rafieepoor, Mohammad Tanhaei, Ghazal Soleymani, Masoumeh Azimirad, Amir Sadeghi, Nasser Ebrahimi Daryani, Farhad Zamani, Ramin Talaei, Abbas Yadegar, Seyed Reza Mohebi, Ghazal Sherkat, Mehrdad Hagh Azalli, Habib Malekpour, Gholamreza Hemmasi, Mohammad Reza Zali

**Affiliations:** a Foodborne and Waterborne Diseases Research Center, Research Institute for Gastroenterology and Liver Diseases, Shahid Beheshti University of Medical Sciences, Tehran, Iran; b Basic and Molecular Epidemiology of Gastrointestinal Disorders Research Center, Research Institute for Gastroenterology and Liver Diseases, Shahid Beheshti University of Medical Sciences, Tehran, Iran; c Department of Microbiology and Microbial Biotechnology, Faculty of Life Sciences and Biotechnology, Shahid Beheshti University, Tehran, Iran; d Gastroenterology and Liver Diseases Research Center, Research Institute for Gastroenterology and Liver Diseases, Shahid Beheshti University of Medical Sciences, Tehran, Iran; e Department of Gastroenterology and Hepatology, Tehran University of Medical Sciences, Tehran, Iran; f Gastrointestinal and Liver Diseases Research Center, Iran University of Medical Sciences, Tehran, Iran; g Department of Gastroenterology and Hepatology, School of Medicine, Shahid Modarres Hospital, Shahid Beheshti University of Medical Sciences, Tehran, Iran; h Faculty of Mashhad Branch, Islamic Azad University, Mashhad, Iran; i Rajaie Cardiovascular Medical and Research Center, Tehran, Iran; Keck School of Medicine of the University of Southern California

**Keywords:** gastrointestinal endoscopes, health care-associated infections, microbial contamination, microbiological surveillance, multidrug resistance

## Abstract

An alarmingly increasing number of outbreaks caused by contaminated gastrointestinal (GI) endoscopes are being reported as a particularly concerning issue. This study is the first large-scale multicenter survey to evaluate the contamination of GI endoscopes in Tehran, Iran. This multicenter study was conducted among 15 tertiary referral and specialized gastrointestinal settings. Reprocessed GI endoscopes were sampled by the sequence of the flush-brush-flush method. Bacterial and viral contamination, as well as antimicrobial resistance, were explored by culture and molecular assays. A total of 133 reprocessed and ready-to-use GI endoscopes were investigated. In phase I and phase II, 47% and 32%, respectively, of the GI endoscopes were determined to be contaminated. GI flora was the most prevalent contaminant isolated from GI endoscopes, in which the most predominant bacteria were Pseudomonas aeruginosa, Escherichia coli, and Klebsiella pneumoniae, in both phase I and II evaluations. The majority of the isolated bacteria in the current study were considered multidrug-resistant organisms (MDROs). More importantly, we recovered carbapenem-resistant nonfermentative Gram-negative bacilli (CRNFGNB), carbapenem-resistant *Enterobacterales* (CRE), extended-spectrum β-lactamase (ESBL)-producing *Enterobacterales* (ESBL-E), multidrug-resistant Clostridioides difficile, vancomycin-resistant *Enterococcus* (VRE), and drug-resistant *Candida* spp. Disconcertingly, our molecular assays revealed contamination of some reprocessed GI endoscopes with hepatitis B virus (HBV), hepatitis C virus (HCV), and even HIV. This multicenter study indicates a higher-than-expected contamination rate among reprocessed and ready-for-patient-use GI endoscopes, which suggests a higher-than-expected endoscopy-associated infection (EAI) risk, and potentially, morbidity and mortality rate, associated with endoscopy procedures in Tehran, Iran.

**IMPORTANCE** In the light of severe outbreaks caused by multidrug-resistant microorganisms due to contaminated GI endoscopes, understanding to what extent GI endoscopes are inadequately reprocessed is crucial. Several studies assessed contamination of GI endoscopes with various outcomes across the world; however, the prevalence and risk factors of contaminated GI endoscopes and potential subsequent nosocomial spread are still unknown in Iran. The present study is the first large-scale multicenter survey to evaluate the microbial contamination of repossessed and ready-to-use GI endoscopes in Tehran, Iran. Our study showed a higher-than-expected contamination rate among reprocessed GI endoscopes, which suggests potential seeding of deadly but preventable outbreaks associated with endoscopy procedures in Iran. These results suggest that the current reprocessing and process control guidelines do not suffice in Iran. The current study is of particular importance and could provide insights into unrecognized and unidentified endoscopy-associated outbreaks in Iran.

## INTRODUCTION

Contaminated flexible gastrointestinal (GI) endoscopes and accessories have frequently been linked to outbreaks of health care-associated infections worldwide (1). According to the report, Preventable Tragedies, 32 endoscope-associated outbreaks involving almost 400 patients were identified in both academic literature and the popular press that occurred in the United States between January 2000 and December 2017 (2). A systematic review conducted by Kwakman et al. estimated that one in every 1.8 million endoscopies leads to an endoscopy-associated infection (EAI) (3). However, it is conceivable that this simple calculation underestimates the actual incidence of EAIs (“tip of the iceberg”), and the definite rate of transmission of the infections and the corresponding burden of outbreaks is difficult to achieve (4). More importantly, an alarmingly increasing number of outbreaks of multidrug-resistant (MDR) infectious pathogens, known as “superbugs,” caused by contaminated medical devices are being reported as a particularly concerning issue (5).

Various organizations have described guidelines for proper reprocessing of GI endoscopes consisting of a multistep procedure involving precleaning followed by cleaning, high-level disinfection with further rinsing, and drying (6–8). Breaches in reprocessing techniques, the use of damaged endoscopes, and contaminated automated endoscope reprocessors (AERs) could all threaten the safety of patients undergoing GI endoscopies. However, even accurate control of reprocessing procedures may not guarantee the prevention of the survival and transmission of enteric pathogens (9). Because GI endoscopes have a very complex structure, including fibrotic bundles and multiple long narrow tubular channels, as well as being reusable, there are many concerns about the possibility of transmission of infectious agents from one patient to another (10, 11). Moreover, the ability of GI and environmental bacteria to produce biofilms in the channels of endoscopes could be considered an important factor in the failure of endoscope reprocessing (9). Literature reviews indicate that Pseudomonas aeruginosa, Klebsiella pneumoniae, and Escherichia coli are the most frequent bacterial agents implicated in EAIs. These resistant bacteria could be transmitted from previous patients through an inadequate reprocessing procedure or environmental contamination before the endoscopy procedure (12).

Prominently, routine microbiological surveillance of GI endoscopes and their related facilities after reprocessing is highly recommended by gastroenterology and endoscopy societies, such as the American Society for Gastrointestinal Endoscopy (ASGE), the European Society of Gastroenterology and Endoscopy Nurses and Associates (ESGENA) committee, the European Society of Gastrointestinal Endoscopy (ESGE), and the Gastroenterological Society of Australia (GESA) (13–15). The prevalence and risk factors of contaminated GI endoscopes and potential subsequent nosocomial spread are still unknown in Iran and require additional investigation. To the best of our knowledge, the present study is the first large-scale multicenter survey to evaluate the microbial contamination of repossessed and ready-to-use endoscopes in Tehran, Iran. We have presented the results of the monitoring of 15 hospitals and health care settings with two-times sampling of GI endoscopes after reprocessing.

## RESULTS

### Summary of the respective GI centers.

Results were sent to the participating endoscopy centers without additional interpretation, and further action was up to the centers and was not documented for the current study, according to the aim of the study. A total of 15 GI endoscopy units of academic tertiary referral medical centers (*n* = 10/15; 66.6%) and private specialized GI centers (*n* = 5/15; 34.4%) took part in this surveillance study, in which 133 GI endoscopes (48 gastroscopes, 37 colonoscopes, 19 duodenoscopes, and 29 linear echoendoscope) and 22 AERs were investigated. GI endoscopes that were out of service were excluded. The median time between sampling at the centers and sample culturing in the Foodborne and Waterborne Diseases Research Center (FWDRC) was 2 h (interquartile range [IQR], 1 to 3). Table 1 provides an overview of the general characteristics of the monitored endoscopy units and reprocessing performance. Concerning the average number of endoscopic procedures per week, 11 (73.3%) centers replied that they performed more than 100 procedures per week. The quality management of endoscope reprocessing and infection surveillance was regularly performed in 5 (33.3%) GI centers by their hospital or an external assessment institution.

**TABLE 1:** General endoscopy unit characteristics and reprocessing performance^*a*^

Hospital	Type of hospital	Endoscopy procedures/wk	Colonoscopy procedures/wk	ERCP procedures/wk	EUS procedures/wk	Nursing staff available in each endoscopy session	Reprocessing time per instrument (min)	Mode of HLD
Hospital I	ATRC	50–100	50–100	10–50	–	3	15–30	Glutaraldehyde
Hospital II	ATRC	10–50	10–50	<10	10–50	4	5–15	Glutaraldehyde
Hospital III	PSC	20	20	Two/mo	5	3	15–30	Glutaraldehyde
Hospital IV	ATRC	>100	>100	–	–	4	5–15	Glutaraldehyde
Hospital V	ATRC	50–100	50–100	4	<10	3	5–15	Glutaraldehyde
Hospital VI	ATRC	10–20	10–20	10–20	10–20	1	5–15	Glutaraldehyde
Hospital VII	ATRC	50–100	50–100	<10	<10	2	5–15	Glutaraldehyde
Hospital VIII	ATRC	>100	50–100	50–100	>100	2	5–15	Glutaraldehyde
Hospital IX	ATRC	50–100	10–50	–	–	2	>30	Glutaraldehyde
Hospital X	PSC	50–100	10–50	<10	<10	2	15–30	Glutaraldehyde
Hospital XI	PSC	30	20	One/mo	One/mo	2	15–30	Glutaraldehyde
Hospital XII	PSC	45	40	<10	<10	1	15–30	Glutaraldehyde
Hospital XIII	ATRC	>100	>100			2	15–30	Glutaraldehyde

a ATRC, academic tertiary referral medical center; PSC, private specialized medical center; ERCP, endoscopic retrograde cholangiopancreatography; EUS, endoscopic ultrasonography; dashes indicates the absence of the procedure at hospitals.

### Prevalence of microbial contaminants in GI endoscopes.

**FIG 1:**
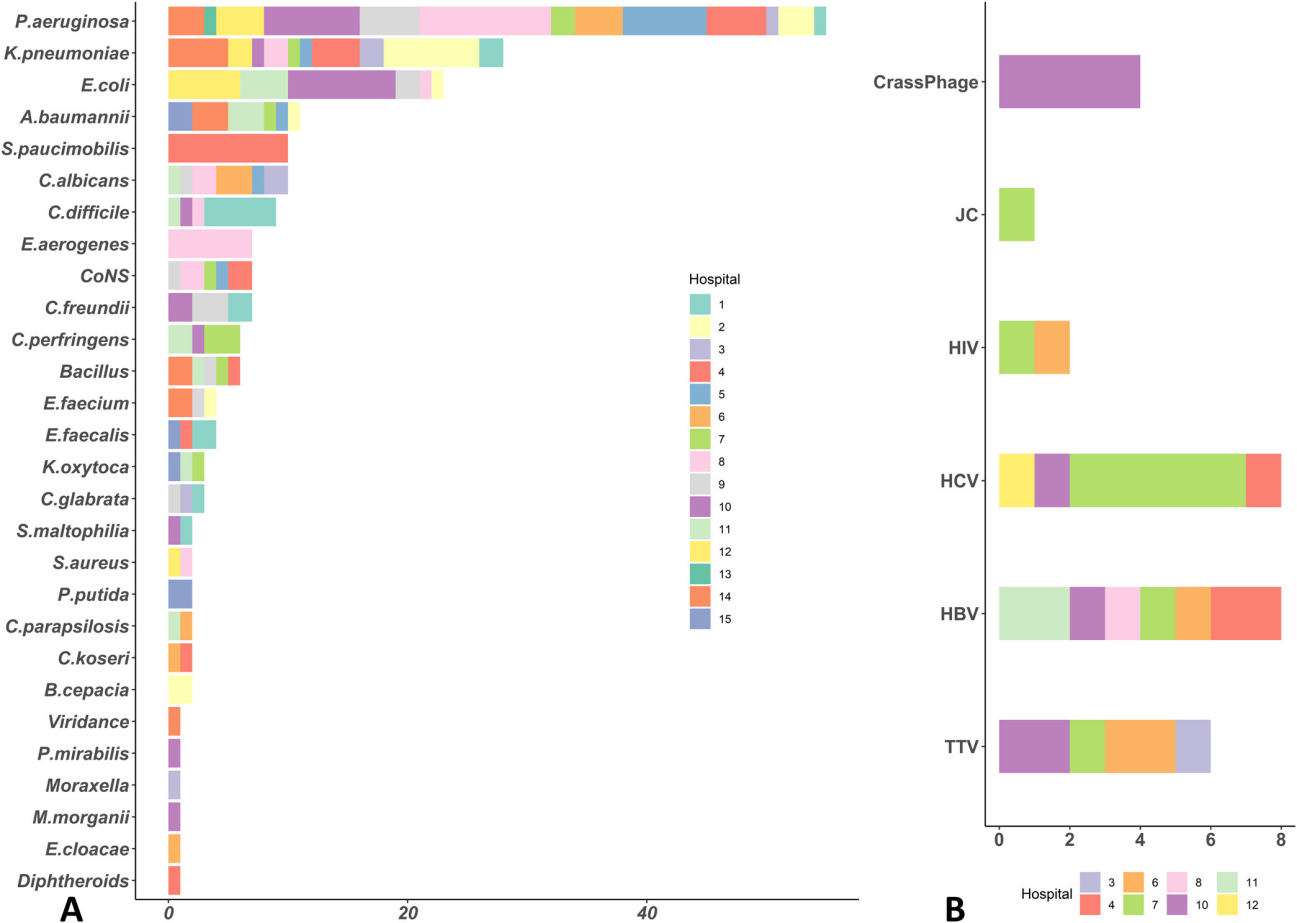

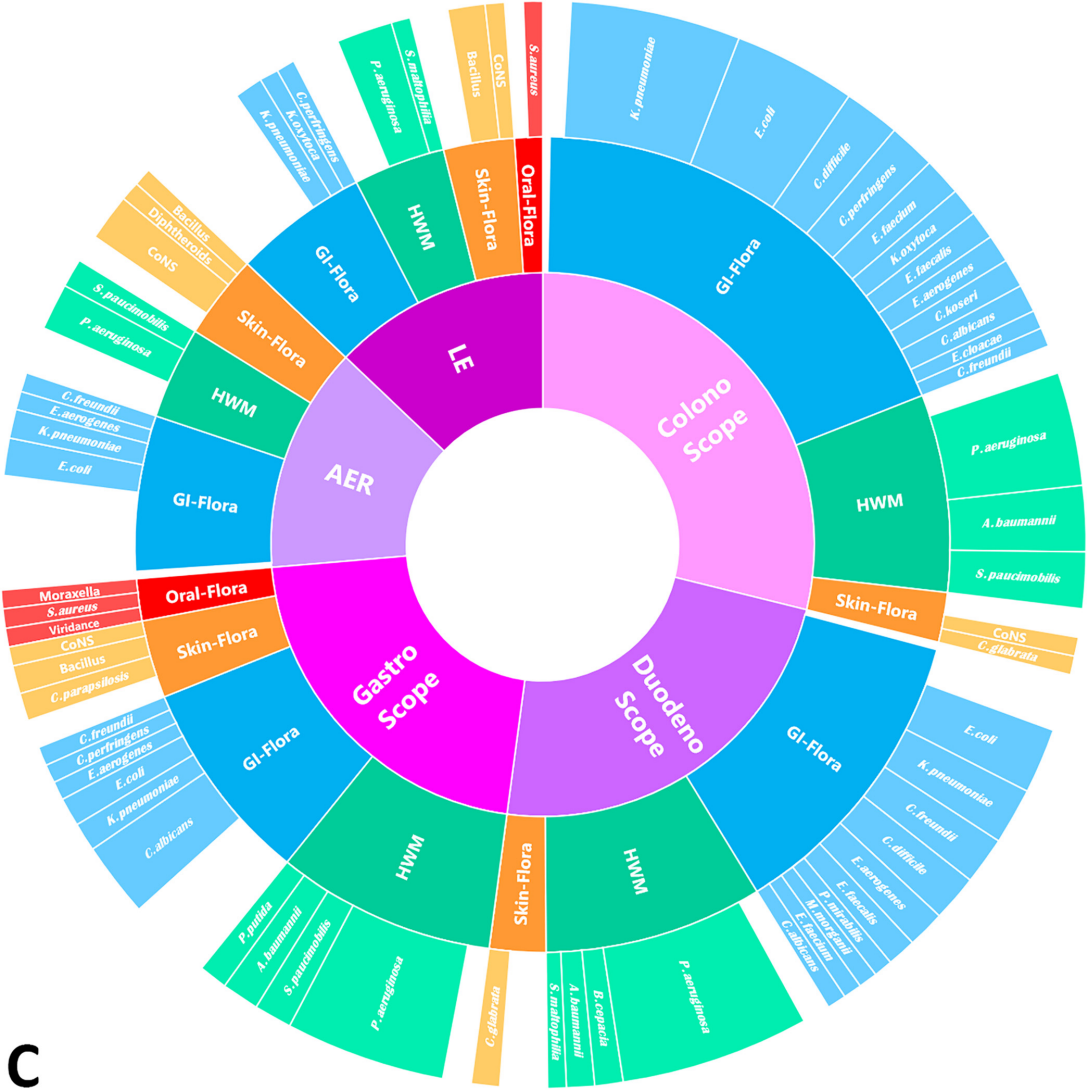
(A and B) The frequency of bacterial and fungal (A) as well as viral (B) contaminants detected in reprocessed GI endoscopes in several hospitals in Tehran, Iran. (C) The prevalence of cultured microbial contaminants according to their origin and the type of GI endoscope. AER, automated endoscope reprocessors; CoNS, coagulase-negative staphylococci; LE, linear echoendoscopes; HWM, hygiene-relevant and waterborne microorganisms.

In phase, I, bacterial and fungal contamination was found in 63/133 (47.4%) reprocessed and ready-to-use GI endoscopes (22 gastroscopes, 26 colonoscopies, 11 duodenoscopes, and 4 linear echoendoscopes), while in phase II, 42/133 (31.6%) GI endoscopes (11 gastroscopes, 19 colonoscopes, 7 duodenoscopes, and 5 linear echoendoscopes) were determined to be contaminated. Colonoscopes yielded markedly more microbial growth than other GI endoscopes in both phases I and II, with a contamination rate of 66.6% and 48.7%, respectively. Among the 19 elevator channel swab samples from duodenoscopes, 11 and 6 yielded microbial growth in phase I and II, respectively, originating from 7 centers across Tehran. The evaluation of 22 AERs yielded microbial growth from 8 (36.4%) swab samples in phase I and 3 (13.6%) in phase II. Surprisingly, three high-level disinfectant (HLD) solution samples (two samples in phase I and one sample in phase II) were microbiologically contaminated in our survey. Generally, no difference was found in microbial contamination prevalence between academic tertiary medical centers and private specialized hospitals (*P = *0.10). The highest contamination rates were found in hospitals IV and VIII. Moreover, most GI endoscopes were contaminated with two or more bacteria, in some cases up to four different bacterial species, and nine cases indicated cocontamination of bacterial and fungal strains. GI flora (51%) were the most prevalent contaminants isolated from GI endoscopes, followed by hygiene-relevant and waterborne microorganisms (HWMO) (35%), in which the most prevalent bacteria were P. aeruginosa (22%), K. pneumoniae (12%), and E. coli (10%) in both phase I and II evaluations (Fig. 1). Table S1 in the supplemental material presents the details of contamination prevalence of the GI endoscopes and AERs as well as different microorganisms that were isolated.

### Detection of drug-resistant superbugs and transmissible resistance genes.

Phenotypic and genotypic antibiotic susceptibility tests were performed on recovered bacterial and fungal isolates. In this study, we focused on the detection of carbapenem-resistant nonfermentative Gram-negative bacilli (CRNFGNB), carbapenem-resistant *Enterobacterales* (CRE), extended-spectrum β-lactamase (ESBL)-producing *Enterobacterales* (ESBL-E), MDR Clostridioides difficile, vancomycin-resistant *Enterococcus* (VRE), methicillin-resistant S. aureus (MRSA), and drug-resistant *Candida* spp. The majority of the isolated bacteria in the current study were considered multidrug-resistant organisms (MDROs). More importantly, we recovered 3 extensively drug-resistant (XDR) Acinetobacter baumannii, 11 MDR and carbapenem-resistant P. aeruginosa, 9 MDR C. difficile, 3 VREs, 4 CREs (including two K. pneumoniae carbapenemase [KPC]-producing and two New Delhi metallo-β lactamase (NDM)-producing Klebsiella), 39 ESBL-producing *Enterobacterales*, and 2 MRSA strains. Table 2 indicates critical drug-resistant superbugs and the relevant transmissible resistance genes.

**TABLE 2:** Prevalence of drug-resistant superbugs and the related genes detected from GI endoscope samples

Antibiotic-resistant threat	Prevalence ratio [*n* (%)]	Related transposable resistance gene(s)	Source(s)	Location
Carbapenem-resistant Acinetobacter baumannii (CRAB)	6/133 (3.7)	*OXA-23*, *OXA-24*, *OXA-58*, *VIM*	Colonoscopes, duodenoscopes, gastroscopes	Hospitals II, XI, XV
Carbapenem-resistant Pseudomonas aeruginosa (CRPA)	11/133 (8.3)	*IMP*, *VIM*, *GES*, *SIM*, *KPC*	Colonoscopes, duodenoscopes, gastroscopes, AERs	Hospitals IV, V, VIII, IX, XII
Carbapenem-resistant *Enterobacteriaceae* (CRE)	4/133 (3)	*KPC*, *NDM*	Colonoscopes, linear echoendoscopes	Hospitals IV, VII, XIV
ESBL-producing *Enterobacteriaceae* (EPE)	39/133 (29.3)	*CTX-M*, *TEM*, *SHV*, *VEB*	Colonoscopes, duodenoscopes, gastroscopes, linear echoendoscopes, AERs	Hospitals I, II, III, IV, VI, VII, VIII, IX, X, XI, XII, XIV, XV
MDR Clostridioides difficile (*tcdA^+^, tcdB^+^*)	9/133 (6.8)	*vanG*, *ermB*	Colonoscopes, duodenoscopes	Hospitals I, VIII, X, XI
Vancomycin-resistant enterococci (VRE)	3/133 (2.2)	*vanA*	Colonoscopes	Hospitals IV, XIV, XV
Methicillin-resistant Staphylococcus aureus (MRSA)	2/133 (2.2)	*mecA*	Gastroscope, linear echoendoscopes	Hospitals VIII, XII
MDR *Candida*	5/133 (3.7)	ND^*a*^	Colonoscopes, duodenoscopes, gastroscopes	Hospitals I, III, VI

a ND, not-determined.

### Viral contaminants in GI endoscopes.

**FIG 2:**
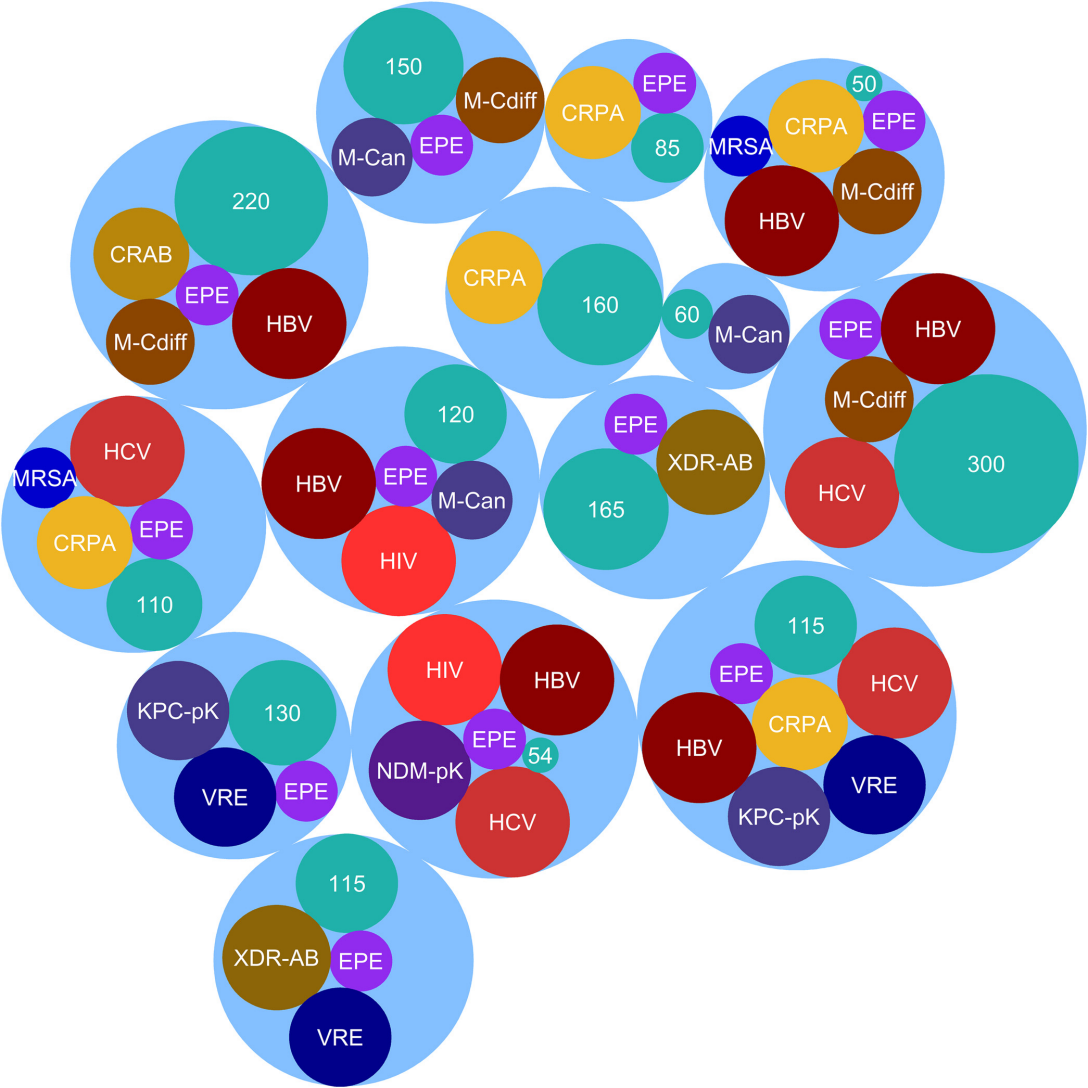
Major superbugs and viral pathogens detected from the reprocessed and ready-to-use GI endoscopes in 15 leading GI endoscopy centers in Tehran, Iran. Each blue circle shows an individual hospital, and the numbers indicate the number of endoscopy procedures per week in the related center. CRPA, carbapenem-resistant P. aeruginosa; EPE, ESBL-producing *Enterobacteriaceae*; M-Can, MDR *Candida*; M-Cdiff, MDR C. difficile; MRSA, methicillin-resistant S. aureus; KPC-pK, KPC-producing Klebsiella; NDM-pK, NDM-producing Klebsiella; VRE, vancomycin-resistant *Enterococci*; XDR-AB, XDR A. baumannii.

Importantly, in the current study, molecular assays revealed contamination of reprocessed GI endoscopes with viral pathogens, including hepatitis B virus (HBV), hepatitis C virus (HCV), and even HIV (Table S1). Critically, HBV contamination was detected in 8 GI endoscopes from 6 hospitals. Moreover, 8 GI endoscopes sampled from 4 hospitals were found to be contaminated with HCV. Impressively, we found seriously alarming HCV contamination in GI endoscopes applied in hospital VII. Additionally, we detected the HIV genome in two GI endoscopes from two different hospitals. Fig. 2 illustrates major superbugs and pathogenic viruses detected in the included hospitals.

## DISCUSSION

To our knowledge, this is the first multicenter report that describes the prevalence of microbial contamination in GI endoscopes in Iran to date. In this study, unfortunately, we found that approximately half of the reprocessed GI endoscopes were microbiologically contaminated, with 93.3% (14/15) of the GI endoscopy centers having at least one contaminated endoscope intended to be ready for patient use. Importantly, it should be noted that these 15 hospitals and health care settings correspond to more than 80% of total endoscopy procedures in Tehran and more than 40% of endoscopy procedures in Iran. Our findings indicate the risk of transmission of resistant superbugs and even viral pathogens to patients undergoing GI endoscopic procedures and suggest that several unrecognized and underreported EAI outbreaks may be occurring in Iran. This study demonstrates the need for immediate and considerable attention by health care facilities and public health authorities in Iran to minimize the risk of outbreaks of superbugs and viral pathogens related to GI endoscopy because of the severity of these infections and limited treatment options. Such outbreaks have been previously reported with a significant mortality rate. For example, Wendorf et al. reported an outbreak of AmpC-producing E. coli infections resistant to third-generation cephalosporins and carbapenems among patients with complicated pancreatic and biliary disease undergoing endoscopic retrograde cholangiopancreatography (ERCP) at a hospital in the United States between November 2012 and August 2013, with a mortality rate of 56% at 30 days (16).

The rates of GI endoscope contamination (47.4%) in this study are relatively higher than in previous studies reported from other countries. In a nationwide study, Rauwers et al. reported that 22% of reprocessed duodenoscopes originating from 26 (39%) centers in the Netherlands were microbiologically contaminated (17). In a microbiological surveillance study of GI endoscopes in France, Saliou et al. reported that 264 of 762 (34.6%) gastroscopes and colonoscopes yielded microbial growth (>25 CFU/mL), which showed a higher level of contamination than the target (18). Some studies have revealed a significantly lower degree of GI endoscope contamination. For instance, in a study performed by Decristoforo et al. in Austria, the microbial contamination rate of GI endoscopes was 1.3% to 4.6% according to the national guideline, suggesting the high quality of endoscope reprocessing, drying, and storage in this country (19). Possible reasons for the different contamination rates in these studies could be due to the different guidelines applied for precleaning, cleaning, disinfection, drying, and storage in endoscopy centers. The factors that influence the adequacy of the reprocessing and disinfection process include endoscope maintenance and repair services issues (20, 21), human factors (22), breaches in reprocessing equipment (23), and inadequate drying before storage (24). We postulate that inadequate and ineffective reprocessing of GI endoscopes in the respective GI centers in our study results in a high contamination rate. Another contributing factor is conducting regular microbiological surveillance at health care facilities as well as the use of different approaches applied for sampling, microbial monitoring, and result interpretation. Previous studies have confirmed that postprocedure or daily microbiological surveillance leads to remarkably lower contamination rates in GI centers over time (25, 26). This could be attributed to the fact that continuous feedback on microbial monitoring resulted in considerable attention, achieving lower contamination rates. According to the current national awareness, it is not common practice to perform microbial contamination surveillance in GI endoscopy centers in Iran, particularly no daily or postprocedure monitoring, as national guidelines do not request these.

The type of contaminant agent can provide an indication of where the endoscope cleaning and reprocessing procedure is faulty. In the current study, GI flora, particularly *K. pneumonia* and E. coli, were the most common contaminants of GI endoscopes, indicating the presence of organic debris (e.g., blood, gastroenteric secretions, and feces) originating from previous patients. Indeed, during GI endoscopy, endoscopes are exposed to the gut flora, and thus, contamination of reprocessed endoscopes with enteric flora indicates breaches in reprocessing protocols and infection control practices. Enteric microorganism contamination could be due to technical problems that existed in the reprocessing process, AER malfunction, or the presence of endoscope damage or occur because the reprocessing procedure is not performed adequately. Disastrously, in this study, we recovered MDR C. difficile strains that were *tcdA*^+^/*tcdB*^+^ from colonoscopes and duodenoscopes applied in hospital I. Moreover, we found that the samples collected from GI endoscopes and even the AER used in hospital VIII yielded Enterobacter aerogenes growth. In hospital XII we identified contamination of the reprocessed colonoscopes, AER, and even HLD solution with MDR E. coli. We postulate that these high rates of GI flora contamination may be due to defective and combined reprocessing, resulting in contamination of upper GI endoscopes such as gastroscopes and duodenoscopes and also AERs during the reprocessing procedure with a source of one or more contaminated colonoscopes. Moreover, the use of inferior HLD, long runs for HLD replacement, AER malfunction, and damaged endoscopes and accessories could be considered risk factors for scope-to-scope and subsequently scope-to-person transmission of drug-resistant enteric microorganisms. Importantly, to prevent this efficiently, multiple endoscopes should not be reprocessed in the same AER run, and auto-disinfection of AERs should take place after every reprocessing cycle.

In the present study, a substantial number of GI endoscopes were contaminated with P. aeruginosa, which is considered an HWMO. P. aeruginosa can survive and proliferate in the moist internal channels of GI endoscopes as well as the internal surface of AREs and is able to form biofilms and microcolonies which are extremely difficult to remove from suction/biopsy channels (27). Allen et al. reported that insufficient drying of GI endoscopes after reprocessing was identified as the cause of an ERCP-associated P. aeruginosa outbreak in the United States (28). This high prevalence of P. aeruginosa in our study could imply inadequate final rinsing and/or insufficient drying of endoscopes before storage in the included centers. Moreover, we found a substantial number of GI endoscopes, as well as AERs in hospital IV, contaminated with Sphingomonas paucimobilis. *S. paucimobilis* is a nonfermenting Gram-negative bacilli that is emerging as an opportunistic pathogen and has been isolated from wastewater, water equipment used in hospitals, and drinking water distribution systems (29). This bacterium has been revealed to form biofilms in water piping, and it has been isolated from ultrapure water systems in hospital water and dental water (30, 31). Although this bacterium is not considered to be a major pathogen, several nosocomial bacteremia and peritonitis cases have been reported that were related to *S. paucimobilis* (29). In our view, contamination with HWMOs arises from the use of unfiltered water for reprocessing facilities, as well as improper endoscope handling before and during patient exposure.

The identification of MDRO and superbug-related outbreaks associated with contaminated GI endoscopes, especially duodenoscopes, dates back to at least the 1980s (28). From 1997 to 2015, 433 MDRO infections related to contaminated GI endoscopes were identified and reported, which included 13 deaths (32). In response to these reports, the Centers for Disease Control and Prevention (CDC) alerted professional medical societies such as the ASGE as well as the Food and Drug Administration (FDA) to prompt the investigation and development of action plans. Disconcertingly, in our study, most isolated microorganisms were considered MDRO, which demonstrates the risk of endoscope-associated transmission of MDR infections to exposed patients. More importantly, in the current study, we isolated various XDR A. baumannii, CRNFGNB, CRE, ESBL-E, MDR C. difficile, VRE, MRSA, and MDR *Candida* strains. The lack of information regarding EIA outbreaks in Iran was particularly worrying when we noted that four CRKP harboring KPC and NDM genes were detected in three hospitals. Since there had been no previously reported endoscopy-associated nosocomial outbreaks in Iran, we questioned whether we may have missed such severe outbreaks because the superbugs have been isolated from leading endoscopy centers in Tehran. To our knowledge, this study is the first to report the isolation of KPC and NDM-producing K. pneumoniae strains from active medical devices. Researchers have reported several CRE infection outbreaks linked to contaminated duodenoscopes, including OXA-232-expressing K. pneumoniae (33), NDM-producing E. coli (34), AmpC-producing E. coli (16), KPC-2-producing K. pneumoniae (35), and OXA-48 expressing K. pneumoniae (36). Muscarella previously described that contaminated endoscopes represent an important risk factor for the spread of CRE and their related superbugs, with associated morbidity and mortality following GI endoscopy (37). Therapeutic duodenoscopes and gastroscopes are more prone to transferring severe and fatal infections because of the more invasive procedures carried out with these types of endoscopes (9). Moreover, duodenoscopes possess a mobile elevator channel on their distal tip, which is difficult to clean and frequently reported to be contaminated with the outbreak’s superbug strain (37).

Disastrously, in the current study, molecular-based assays revealed that some GI endoscopes are contaminated with highly infectious and pathogenic viruses, including HBV, HCV, and HIV. These viruses could be easily transmitted through contact of GI endoscopes with the blood or body fluids of a previously infected person. Despite the high infectivity of HBV, there are a few reports from the 1990s that documented endoscopic HBV transmission after such procedures (38–40). The overall risk of HCV transmission through GI endoscopy is controversial; however, some clinical studies indicated that GI endoscopic procedures were associated with HCV infection (41–44). In our surveillance, we detected the viral genome of HBV and HCV from six and four GI endoscopy centers, respectively. Significantly, gastroscopes, colonoscopes, and linear echoendoscopes were contaminated with HCV in hospital VII. It was previously described that the risk of endoscopic transmission of HCV and HBV is low when adequate endoscope reprocessing is applied (45). No cases of HIV transmission attributed to GI endoscopic procedures have been reported so far. Hence, our findings are particularly alarming for health care providers because there are several breaches in the GI endoscope reprocessing in referral and specialized GI centers in Iran. This can be much more troubling if we consider the low incidence of acute HBV, HCV, and HIV infections in the general population and their often asymptomatic course.

**Conclusion.** This multicenter study indicates a higher-than-expected contamination rate among reprocessed and ready-for-use GI endoscopes, which suggests a high potential risk of EAI outbreaks in Iran. Our findings highlight the importance of public health surveillance for recognizing microbiologically contaminated GI endoscopes and identifying resistant superbugs as well as viral infections related to endoscopy procedures. Routine and proper surveillance systems should consider the quality of endoscope reprocessing and the training of staff in GI endoscopy centers. In addition, routine endoscope evaluation and maintenance schedules need to be included in the approval process for these devices. GI endoscopy units should withdraw the endoscopes that remained contaminated despite repeated reprocessing because they are usually old and defective, and the wear of channels made their disinfection inefficient. Additionally, it is crucial to promptly recognize outbreaks and monitor and respond to the ongoing threat from MDR organisms in health care facilities. The recognized EAIs should be immediately reported to public health authorities and require public health action.

## MATERIALS AND METHODS

### Study design.

This multicenter, provincewide cross-sectional study was conducted among 15 GI endoscopy centers, including tertiary referral hospitals and private specialized GI clinics in Tehran, Iran, in 2021. The activities involved in this investigation were reviewed and approved by the Research Institute for Gastroenterology and Liver Diseases (RIGLD) affiliated with Shahid Beheshti University of Medical Sciences, Tehran, Iran (project no. IR.SBMU.RIGLD.REC.1399.031). During the study, we invited the centers to sample all reprocessed GI endoscopes present in their endoscopy department twice, one time before the exchange of high-level disinfectant (HLD) used for AERs (phase I) and one time following the exchange of the HLD (phase II). GI endoscopes were eligible for sampling if they were reprocessed with HLD, dried, and ready for patient use. Data about the number of procedures at the center and reprocessing methods were recorded.

### Sampling method.

Reprocessed GI endoscopes were sampled with the sequence of the flush-brush-flush method as recommended by the Gastroenterological Society of Australia and Gastroenterological Nurses College of Australia (GESA-GENCA) (46). Sampling was performed under highly aseptic conditions by two experts while the GI scopes were placed on a sterile surface. Briefly, this method consists of flushing endoscope biopsy/suction channels with 50 mL sterile Dey-Engley neutralizing broth (Himedia Laboratories, India), brushing the biopsy/suction channels using a sterile single-use, dual-ended cleaning brush (Olympus, Hamburg, Germany) to rub the inner lumen (including possible biofilms), and then flushing again with 50 mL of the medium, in both antegrade and retrograde manner. The upper part of the brush was cut off using a sterile scissor and put into a 50-mL aseptic microbiological container. Then 5 mL of the total volume of the flush fluid was inoculated into thioglycolate broth (for anaerobic culture) (Merck, Darmstadt, Germany), and at least 50 mL of the rest of the fluid was collected at the distal tip in the same container used for the testing of the clipped brush. Additionally, a swab was taken from the elevator channels of all duodenoscopes using ESwabs (Copan Italia S.p.A., Brescia, Italy). Moreover, two samples were separately taken from the internal surface of the AER (after a completed cycle of cleaning, high-level disinfection, and final rinsing) and the HLD solution of the AER (1 mL) and collected in sterile tubes. The decision for reprocessing the endoscopes after sampling was up to the respective centers and was not recorded based on the aim of the current study. Fig S1 schematically shows the sampling strategy, bacterial and viral identification, and resistance gene detection in this study.

### Bacterial isolation and antibiotic susceptibility testing.

Bacterial contamination was defined as culture positive with ≥20 CFU/20 mL for hygiene-relevant and waterborne microorganisms (HWMO) or the presence of bacterial strains with GI and/or oral origin, independent of CFU count (17). Samples were immediately processed and cultured on the day of receipt as described below. Flush fluid samples were filtered through a 0.45-μm filter, the filtrate of which was forced on blood agar plates (BAP) (Merck, Darmstadt, Germany). Brush tips and ESwabs were vortexed in their medium, 50 μL of which was poured on BAP and MacConkey medium (Merck), as well as Sabouraud dextrose agar (Merck). The plates were incubated at 35° C and were examined for growth every 24 h for 3 days. Then, 100 μL of the thioglycolate broth was cultured on cycloserine-cefoxitin-fructose agar (CCFA) (Mast Group Ltd., Merseyside, UK) supplemented with 7% sheep blood and tryptose sulfite cycloserine with egg yolk agar (TSC-EYA) for isolation of Clostridioides difficile and Clostridium perfringens, respectively. The cultured plates were incubated under anaerobic conditions (85% N_2_, 10% CO_2_, and 5% H_2_) generated using an Anoxomat gas exchange system (Mart Microbiology BV, Lichtenvoorde, Netherlands) at 37°C for 48 to 72 h. Recovered microorganisms were isolated and identified by standard biochemical tests and API 20E methods (47). Additionally, molecular confirmation of bacterial identification was carried out on the isolated strains using the PCR method, as previously described (48, 49).

All microbial isolates were tested for antimicrobial susceptibilities and interpreted using the Clinical and Laboratory Standards Institute (CLSI) reference (50). Extended-spectrum β-lactamases (ESBLs) and carbapenem resistance phenotypes were examined, as described previously (50–53). MDR organisms (MDROs) were defined as microorganisms resistant to at least three drugs from a variety of antibiotic classes. Extensively drug-resistant (XDR) Acinetobacter baumannii was defined as A. baumannii isolates that were resistant to all active antibiotic categories against the considered microorganism except colistin and tigecycline.

### Molecular detection of major antibiotic resistance.

Multiplex PCR assays were performed to detect the associated genes encoding acquired ESBLs and carbapenemases using specific primers, as previously described with slight modifications (54, 55). Vancomycin-resistant enterococcus (VRE) surveillance was performed with the multiplex PCR assay reported by Kariyama et al. (56). For the molecular detection of methicillin-resistant S. aureus (MRSA), PCR analysis of the *mecA* gene was performed as described previously (57).

### Viral contamination assays.

Molecular detection of some viruses, including torque teno virus (TTV), crAssphage (as human fecal markers), hepatitis B virus (HBV), hepatitis C virus (HCV), human immunodeficiency virus (HIV), John Cunningham virus (JCV), BK virus, and severe acute respiratory syndrome coronavirus 2 (SARS-CoV-2), was performed in all samples. Briefly, the concentration of the virus from the filtered flush fluids was performed with PEG-6000 (polyethylene glycol 6000). Accordingly, 12.5% PEG 6000 and 2.5% NaCl were added to the samples at the final concentration. The eluate was stirred at 4°C overnight and then centrifuged at 15,000 × *g* for 30 min, and the pellet was suspended in 200 μL of phosphate-buffered saline (PBS) and stored at −20° C for subsequent analysis. Viral nucleic acids were extracted from the concentrated viral suspension using a Qiagen viral RNA minikit (Hilden, Germany) according to the instructions. The extracted nucleic acids were examined by nested PCR to diagnose the presence of TTV, as described previously (58). The HBV genome was detected using a seminested PCR method and confirmed using DNA sequencing. Additionally, commercial kits based on the real-time PCR method were used to identify HCV (AmpliSens HCV Monitor-FRT, Amplisence Biotechnologies, Moscow, Russia), HIV (GeneProof HIV type 1 [HIV-1] PCR kit), and SARS-CoV-2 (COVITECH COVID19 multiplex real-time PCR kit, Tehran, Iran).

### Ethics approval and consent to participate.

No ethics approval was required for this study.
