# Erratum for Houri et al., “High Prevalence Rate of Microbial Contamination in Patient-Ready Gastrointestinal Endoscopes in Tehran, Iran: an Alarming Sign for the Occurrence of Severe Outbreaks”

**DOI:** 10.1128/spectrum.00581-23

**Published:** 2023-03-13

**Authors:** Hamidreza Houri, Hamid Asadzadeh Aghdaei, Sepideh Firuzabadi, Babak Khorsand, Fatemeh Soltanpoor, Maedeh Rafieepoor, Mohammad Tanhaei, Ghazal Soleymani, Masoumeh Azimirad, Amir Sadeghi, Nasser Ebrahimi Daryani, Farhad Zamani, Ramin Talaei, Abbas Yadegar, Seyed Reza Mohebbi, Ghazal Sherkat, Mehrdad Hagh Azalli, Habib Malekpour, Gholamreza Hemmasi, Mohammad Reza Zali

## ERRATUM

Volume 10, no. 5, e01897-22, 2022, https://doi.org/10.1128/Spectrum.01897-22. Page 1, byline: Seyed Reza Mohebbi’s name should appear as shown in this Erratum.

Page 3, Table 1, footnote *a*: “indicates” should read “indicate.”

