# Supplementary material for: High Prevalence Rate of Microbial Contamination in Patient-Ready Gastrointestinal Endoscopes in Tehran, Iran: an Alarming Sign for the Occurrence of Severe Outbreaks
Source: Microbiol Spectr. 2022 Sep 29;10(5):e01897-22. doi: 10.1128/spectrum.01897-22 (PMC9602500; doi:10.1128/spectrum.01897-22)
Supplement: Supplemental file 1 — Table S1 and Fig. S1. Download spectrum.01897-22-s0001.pdf, PDF file, 0.2 MB [file spectrum.01897-22-s0001.pdf]

Supplemental Tables.

Table S1. Results of microbiological surveillance of different GI endoscopes types and automated endoscope reprocessors

**Hospital**	**Type of hospital**	**Time of HLD changing (time between Phase I and II)**	**Specimen source (Sample site)**	**Contamination ratio, isolated microorganisms**		**MDRO detection**		**Viral detection**	
				**Phase I**	**Phase II**	**Phase I**	**Phase II**	**Phase I**	**Phase II**
Hospital I	ATRC	12 days	Colonoscopes, *n*=4 (biopsy/suction channel)	50%, C1: *C. difficile* (*tcdA*$^+$, *tcdB*$^+$). C2: *C. difficile* (*tcdA*$^+$, *tcdB*$^+$).	50%, C1: *K. pneumoniae*. C2: *K. pneumoniae*.	Yes	Yes	No	No
			Duodenoscopes, *n*=2 (biopsy/suction channel)	100%, D1: *C. freundii*; *C. difficile* (*tcdA*$^+$, *tcdB*$^+$). D2: *E. faecalis*; *C. difficile* (*tcdA*$^+$, *tcdB*$^+$); *C. glabrata*.	0%, No growth	Yes	No	No	No
			Duodenoscopes, *n*=2 (elevator channel)	100%, D1: *C. freundii*; *S. maltophilia*; *C. difficile* (*tcdA*$^+$, *tcdB*$^+$). D2: *E. faecalis*; *C. difficile* (*tcdA*$^+$, *tcdB*$^+$).	50%, D2: *P. aeruginosa*	Yes	No	No	No
Hospital II	ATRC	5 days	Duodenoscopes, *n*=2 (biopsy/suction channel)	50%, *K. pneumoniae*; *P. aeruginosa*.	50%, *B. cepacia*; *K. pneumoniae*.	Yes	Yes	No	No
			Duodenoscopes, *n*=2 (elevator channel)	100%, D1: *K. pneumoniae*; *P. aeruginosa*. D2: *E. faecium*.	50%, *B. cepacia*; *K. pneumoniae*.	Yes	Yes	No	No
			Colonoscopes, *n*=4 (biopsy/suction channel)	50%, C1: *P. aeruginosa;* XDR-*A. baumannii* [a]. C3: *E. coli*.	50 %, C1: *K. pneumoniae*. C2: *K. pneumoniae*.	Yes	Yes	No	No
			AER, *n*=1 (HLD solution)	100%, *K. pneumoniae*	100%, *K. pneumoniae*.	Yes	Yes	No	No
Hospital III	ATRC	28 days	Colonoscopes, *n*=2 (biopsy/suction channel)	50%, *C. albicans*; *C. glabrata*; *K. pneumoniae*.	0%, No growth	Yes	No	No	No
			Gastroscopes, *n*=2 (biopsy/suction channel)	50%, *C. albicans*; *Moraxella* spp.	100%, G1: *K. pneumoniae*. G2: *P. aeruginosa*.	No	Yes	Yes, TTV	No
Hospital IV	ATRC	28 days	Colonoscopes, *n*=4 (biopsy/suction channel)	100%, C1: *K. pneumoniae*; *S. paucimobilis*. C2: *S. paucimobilis* C3: *P. aeruginosa* [2 different strains]; *K. pneumoniae*; *S. paucimobilis*	75%, C2: *K. pneumoniae*; *S. paucimobilis* C3: *E. faecalis* [VRE] C4: *P. aeruginosa*; *C. koseri*	Yes	Yes	Yes, HBV; HCV.	No

				C4: *K. pneumoniae*; *S. paucimobilis*.					
			Gastroscopes, n=3 (biopsy/suction channel)	100%, G1: *P. aeruginosa*. G2: *P. aeruginosa*; *S. paucimobilis*. G3: *S. paucimobilis*.	33%, G1: *P. aeruginosa*; *S. paucimobilis*.	Yes	Yes	Yes, HBV.	No
			AERs, *n*=2 (swab sample)	100%, AER1: *S. paucimobilis*; CoNS; nondiphtherial-*Corynebacterium* spp. AER2: *Bacillus* spp.	50%, AER1: *S. paucimobilis*; CoNS.	No	No	No	No
Hospital V	ATRC	28 days	Duodenoscopes, *n*=2 (biopsy/suction channel)	50%, *P. aeruginosa*; *A. baumannii*.	50%, *P. aeruginosa*	Yes	No	No	No
			Duodenoscopes, *n*=2 (elevator channel)	50%, *P. aeruginosa*; *K. pneumoniae*.	50%, *P. aeruginosa*	Yes	No	No	No
			Gastroscopes, *n*=3 (biopsy/suction channel)	33.3%, *P. aeruginosa*	33.3%, *C. albicans*; *P. aeruginosa*.	Yes	Yes	No	No
			AERs, *n*=1 (swab sample)	100%, CoNS; *P. aeruginosa*	0%, No growth	Yes	No	No	No
Hospital VI	PSC	14 days	Gastroscopes, *n*=4 (biopsy/suction channel)	75%, G1: *C. albicans*. G2: *C. albicans*; *P. aeruginosa*. G3: *P. aeruginosa*; *C. albicans*.	25%, *C. parapsilosis*	Yes	No	No	No
			Colonoscopes, *n*=4 (biopsy/suction channel)	25%, *P. aeruginosa*; *E. cloacae*.	25%, *P. aeruginosa*; *C. koseri*.	Yes	Yes	Yes, HIV (180 GC/ml); TTV.	Yes, HBV; TTV.
Hospital VII	ATRC	14 days	Colonoscopes, *n*=2 (biopsy/suction channel)	100%, C1: *C. perfringens*. C2: *C. perfringens*.	0%, No growth.	No	No	Yes, HBV; HCV; JC.	No
			Gastroscopes, *n*=2 (biopsy/suction channel)	100%, G1: *P. aeruginosa*. G2: *P. aeruginosa*.	50%, *A. baumannii*.	No	Yes	Yes, HCV; TTV.	Yes, HCV; HIV (430 GC/ml)
			Linear echoendoscopes, *n*=2 (biopsy/suction channel)	50%, *K. pneumoniae*; *K. oxytoca*; *C. perfringens*.	50%, *Bacillus* spp; CoNS.	Yes	No	Yes, HCV	Yes, HCV
Hospital VIII	PSC	14 days	Colonoscopes, *n*=2 (biopsy/suction channel)	100%, C1: *K. pneumoniae*; *P. aeruginosa*; *E. aerogenes*; CoNS.	100%, C1: *E. coli*; *P. aeruginosa*. C2: *P. aeruginosa*; *C. difficile* (*tcdA*$^{+}$, *tcdB*$^{+}$).	Yes	Yes	No	No

				C2: *P. aeruginosa* [2 different strains]; *E. aerogenes*.					
			Gastroscopes, *n*=3 (biopsy/suction channel)	100%, G1: *P. aeruginosa.* G2: *C. albicans*; *E. aerogenes*; CoNS.	50%, *C. albicans.*	Yes	No	Yes, HBV	No
			Linear echoendoscopes, *n*=1 (biopsy/suction channel)	100%, *K. pneumoniae*; *S. aureus*.	100%, *P. aeruginosa*.	Yes	Yes	No	No
			Duodenoscopes, *n*=1 (biopsy/suction channel)	100%, *P. aeruginosa*; *E. aerogenes*.	100%, *P. aeruginosa*.	Yes	Yes	No	No
			Duodenoscopes, *n*=1 (elevator channel)	100%, *P. aeruginosa*; *E. aerogenes*.	100%, *P. aeruginosa*; *E. aerogenes*.	Yes	Yes	No	No
			AERs, *n*=1 (swab sample)	100%, *P. aeruginosa*; *E. aerogenes*.	0%, No growth	Yes	No	No	No
Hospital IX	PSC	14 days	Colonoscopes, *n*=3 (biopsy/suction channel)	33.3%, *P. aeruginosa*; *E.coli*; *C. freundii*.	33.3%, *E.coli*; *E. faecium.*	Yes	Yes	No	No
			Gastroscopes, *n*=4 (biopsy/suction channel)	50%, G1: *P. aeruginosa.* G2: *C. freundii*.	0%, No growth	Yes	No	No	No
			Duodenoscopes, *n*=1 (biopsy/suction channel)	100%, *P. aeruginosa*; *C. glabrata*.	100%, *C. albicans*	No	No	No	No
			Duodenoscopes, *n*=1 (elevator channel)	100%, *P. aeruginosa*	0%, No growth	No	No	No	No
			Linear echoendoscopes, *n*=1 (biopsy/suction channel)	0%, No growth	100%, *Bacillus* spp.	No	No	No	No
			AERs, *n*=1 (swab sample)	100%, *P. aeruginosa*; *C. freundii*; CoNS.	0%, No growth.	Yes	No	No	No
Hospital X		25 days	Colonoscopes, n=4 (biopsy/suction channel)	50%, C1: *E.coli; C. difficile.* C2: *E.coli*; *K. pneumoniae*.	50%, C1: *E.coli.* C4: *C. perfringens*.	Yes	No	No	No
			Duodenoscopes, *n*=4 (biopsy/suction channel)	75%, D1: *E.coli*; *P. mirabilis.* D2: *E.coli*; *P. aeruginosa*. D3: *E.coli*; *P. aeruginosa*.	75%, D2: *C. freundii*. D3: *E.coli*; *M. morganii*. D4: *P. aeruginosa*.	Yes	Yes	Yes, HBV; HCV; TTV; Crass Phage.	Yes, Crass Phage.
			Duodenoscopes, *n*=4 (elevator channel)	75%, D1: *E.coli*. D2: *P. aeruginosa*. D3: *E.coli*; *P. aeruginosa*.	50%, D2: *C. freundii*. D4: *P. aeruginosa*.	Yes	Yes	No	No
			Linear echoendoscopes, *n*=6 (biopsy/suction channel)	16.7%, *P. aeruginosa*.	16.7%, *P. aeruginosa; S. maltophilia*.	Yes	Yes	Yes, TTV; Crass Phage.	Yes, Crass Phage.
Hospital XI	ATRC		Colonoscopes, n=3	100%,	33.3%,	Yes	Yes	No	No

			(biopsy/suction channel)	C1: *E.coli*; *C. difficile*.<br>C2: *E.coli*; *A. baumannii*.<br>C3: *C. albicans*; *K. oxytoca*.	*C. perfringens; A. baumannii*.				
			Gastroscopes, *n*=3 (biopsy/suction channel)	66.7%,<br>G1: *E.coli* [two different strains]; *Bacillus* spp.<br>G2: *C. perfringens*.	33.3%,<br>*E.coli*; C. *parapsilosis*	Yes	No	Yes, HBV	No
			Duodenoscopes, *n*=1 (biopsy/suction channel)	100%,<br>*A. baumannii*.	No growth.	Yes	No	Yes, HBV	No
Hospital XII	ATRC	30 days	Colonoscopes, n=2 (biopsy/suction channel)	100%,<br>C1: *E.coli* [two different strains].<br>C2: *E.coli*; *P.* aeruginosa; *K. pneumoniae*.	50%,<br>*E.coli*.	Yes	Yes	Yes, HCV	No
			Gastroscopes, *n*=2 (biopsy/suction channel)	100%,<br>G1: *P. aeruginosa*.<br>G2: *P. aeruginosa*; *K. pneumoniae*.	100%,<br>*P. aeruginosa*; *S. aureus*.	Yes	No	No	No
			AERs, *n*=1 (swab sample)	100%,<br>*E.coli*.	100%,<br>*E.coli*.	Yes	Yes	No	No
			AERs, *n*=1 (HLD solution)	100%,<br>*E.coli*.	0%,<br>No growth.	Yes	No	No	No
Hospital XIII	PSC	14 days	AERs, *n*=2 (swab sample)	50%,<br>*P. aeruginosa*;	0%,<br>No growth.	No	No	No	No
Hospital XIV	PSC	28 days	Colonoscopes, n=3 (biopsy/suction channel)	100%,<br>C1: *K. pneumoniae* [two different strains].<br>C2: *K. pneumoniae*; *E. faecium* [VRE].<br>C3: *K. pneumoniae*; *E. faecium*.	66.6%,<br>C1: *K. pneumoniae*; *A. baumannii*.<br>C2: *K. pneumoniae*.	Yes	Yes	No	No
			Gastroscopes, *n*=4 (biopsy/suction channel)	50%,<br>G1: *P. aeruginosa*.<br>G2: *A. baumannii*.	50%,<br>G1: Viridance Streptococci.<br>G2: *A. baumannii*; *Bacillus* spp.	Yes	Yes	No	No
			Linear echoendoscopes, *n*=1 (biopsy/suction channel)	100,<br>E1: *P. aeruginosa*.	100,<br>E1: *P. aeruginosa*; *Bacillus* spp.	No	No	No	No
Hospital XV	PSC	10 days	Colonoscopes, n=3 (biopsy/suction channel)	33.3%,<br>*E. faecalis* [VRE]; *K. oxytoca*.	66.6%,<br>C1: *A. baumannii* [XDR].<br>C2: *A. baumannii* [XDR].	Yes	Yes	No	No
			Gastroscopes, *n*=4 (biopsy/suction channel)	50%,<br>G1: *P. putida*.<br>G2: *P. putida*.	0%,<br>No growth.	No	No	No	No

[a] XDR-*A. baumannii*: an extensively drug-resistant (XDR) *Acinetobacter baumannii* presented in this sample which was resistant to all antibiotics (all active antibiotic categories against considered microorganisms), except colistin and tigecycline.

ATRCs, academic tertiary referral medical centres; ERCP, endoscopic retrograde cholangiopancreatography; HLD, high-level disinfectant; MRO, multidrug-resistant organism(s); PSCs, private specialized medical centres.

Supplemental Figures.

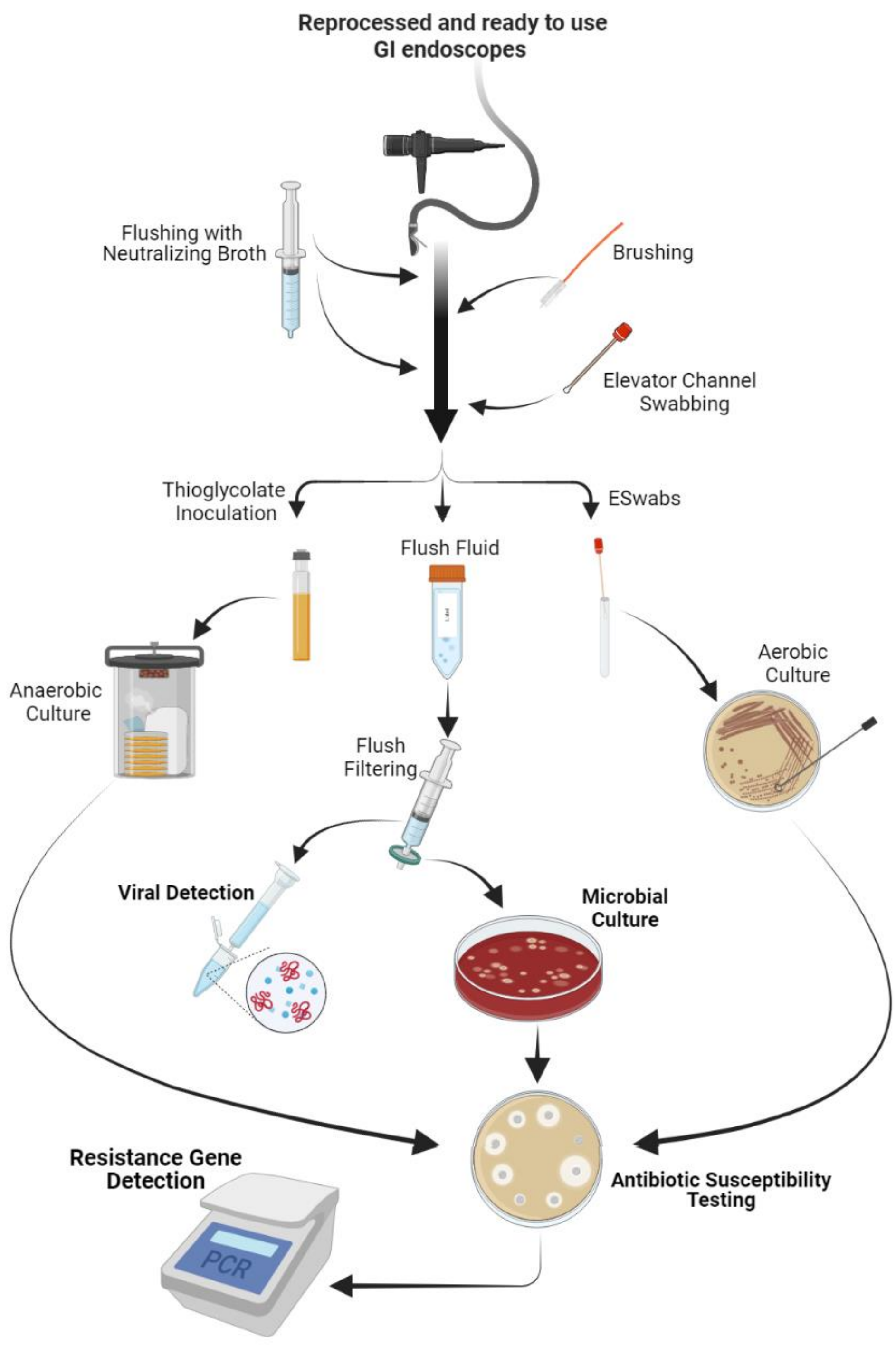

Figure S1. Flow diagram indicating sampling strategy, bacterial and viral identification, and resistance genes detection in this study.
